# Autochthonous Human *Babesia divergens* Infection, England

**DOI:** 10.3201/eid3010.240866

**Published:** 2024-10

**Authors:** Guillermo A. Zabala, Robert Lever, Xin Hui Chan, Henrietta Bristowe, Emer Kilbride, David Richards, Mark Daly, Michael Brown, Nick Johnson, Laura Eve Nabarro, Hanif Esmail, Gauri Godbole, Peter L. Chiodini

**Affiliations:** Infectious Diseases Data Observatory, Oxford, UK (G.A. Zambala);; Hospital for Tropical Diseases, University College Hospital, London, UK (G.A. Zabala, R. Lever, X.H. Chan, H. Bristowe, E. Kilbride, M. Brown, L.E. Nabarro, H. Esmail, G. Godbole, P.L. Chiodini);; London School of Hygiene and Tropical Medicine, London (R. Lever, M. Brown, G. Godbole, P.L. Chodini);; Centre for Tropical Medicine and Global Health, University of Oxford, Oxford (X.H Chan);; North Devon District Hospital, Barnstaple, UK (D. Richards, M. Daly);; Animal and Plant Health Agency, Surrey, UK (N. Johnson);; Institute for Global Health and Medical Research Council Clinical Trials Unit, University College London, London (H. Esmail)

**Keywords:** Babesiosis, parasites, vector-borne infections, ticks, communicable diseases, United Kingdom, England, Babesia, hemolysis, emerging communicable diseases, public health, parasitology

## Abstract

We describe a case of autochthonous human *Babesia divergens* infection in an immunocompetent woman in England. The patient had fever, hemolysis, multiorgan failure, and 18% parasitemia. We confirmed *B. divergens* by 18S rDNA PCR and sequencing. Clinicians should consider babesiosis as a differential diagnosis in patients with unexplained hemolysis.

Babesiosis is caused by intraerythrocytic protozoa from the genus *Babesia*. First reported in 1957 (*1*), babesiosis is now described worldwide. *Babesia* infecting humans come from 4 clades (*2*): 3 clades of small *Babesia*, 1 including *B. microti*, which exists as a species complex; 1 including *B. duncani*; and 1 including *B. divergens*, which, despite being small, is related to the 1 clade of large *Babesia* spp., which infects ungulates. *Ixodes* spp. ticks transmit *Babesia* between animal hosts. Each *Babesia*–vector–mammal host system has its own characteristics, and the ecology and bionomics of the vector tick define the pattern of risk for the human population. Humans are accidental hosts and can also acquire babesiosis by horizontal transmission in blood products and, in rare instances, via transplacental transmission (*3*).

Most human babesiosis cases are caused by *B. microti* species complex or *B. divergens*, but as recognition of human cases increases, other species, some newly described, have been found in humans. *B. microti* is endemic in the Northeast and northern Midwest United States, and ≈2,000 human *B. microti* babesiosis cases are reported annually (*4*). However, cases of *B. divergens* infections are rare, ≈50 reports in the literature, and often cause more severe illness (*5*).

In the United Kingdom, increasing *Babesia* spp. prevalence in *Ixodes* ticks has been noted (*6*), but only 1 human case of *B. divergens* babesiosis has been reported, from Scotland in 1979 (*7*). We describe a case of autochthonous *B. divergens* infection in England.

## The Study

A 72-year-old retired nurse was admitted to a hospital in southwest England after 4 days of fever, body aches, loin pain, and frank hemoglobinuria. She received ciprofloxacin in primary care the preceding day for presumed urinary tract infection, but vomiting and jaundice subsequently developed. Physical examination confirmed fever (>40°C), tachycardia, and jaundice, but no other findings. Hemoglobin was 75 g/L and bilirubin 190 μmol/L.

**Figure 1:**
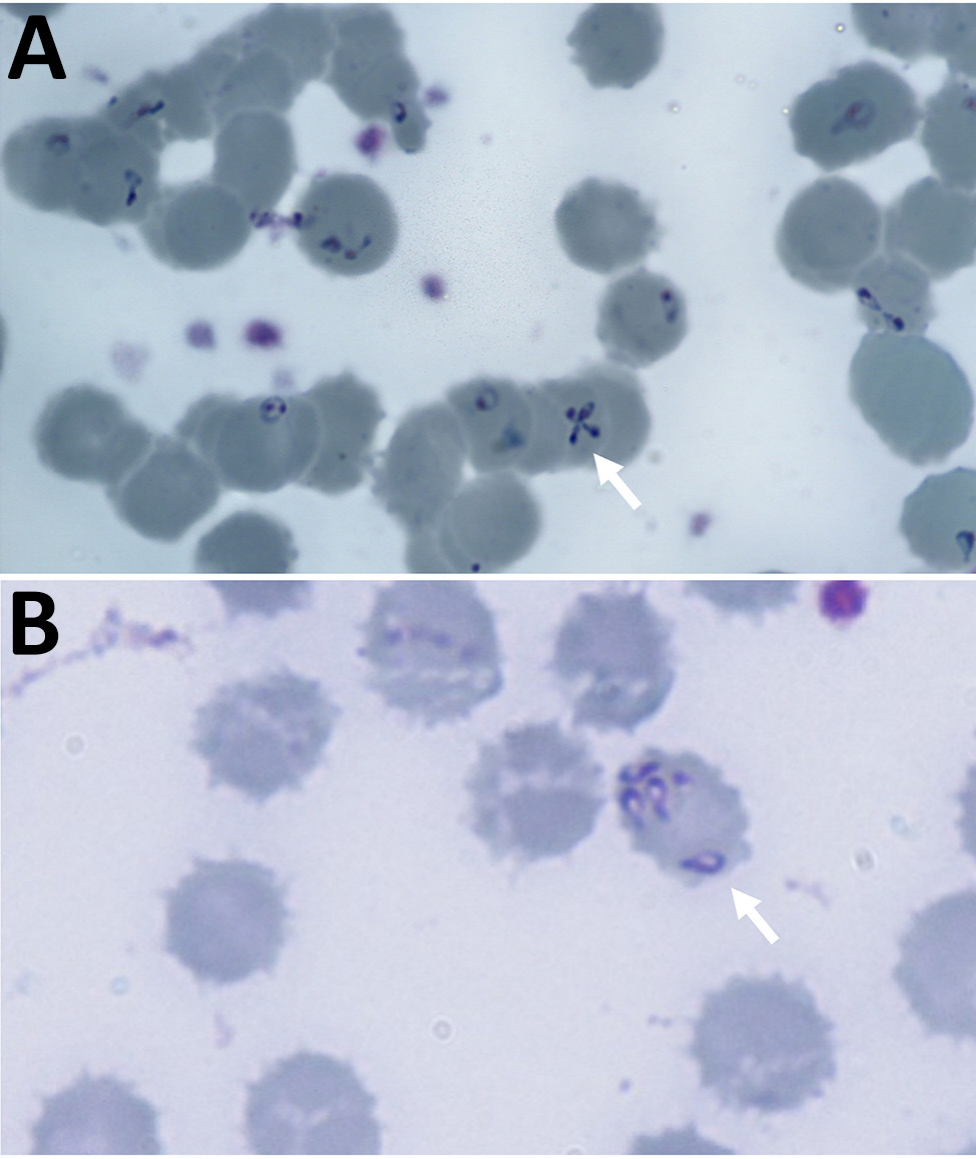
Giemsa-stained thin blood film from a case of autochthonous human *Babesia divergens* infection, England. A) Stains show characteristic Maltese cross form (arrow) and erythrocytes containing 5 pyriform rings. Original magnification ×1,000. B) Absence of pigment in erythrocytes (arrow). Original magnification ×1,000.

Blood film showed intraerythrocytic parasites with the Maltese cross formation, pathognomonic for *Babesia* spp. (Figure 1). We published that morphology as an update to raise awareness in hematology laboratories (*8*). Peripheral parasitemia in erythrocytes was 18% at diagnosis (Table). After consulting the Hospital for Tropical Diseases (HTD), we began treatment with intravenous clindamycin (600 mg every 6 h) and quinine (10 mg/kg every 8 h). One day into admission the patient deteriorated, had severe hypoxia requiring intubation, and was transferred by helicopter to the HTD intensive care unit (ICU).

**Table:** Course of parasitemia during admission and interventions used in a case of autochthonous human *Babesia divergens* infection, England*

Days after admission	% Parasitemia†	Intervention
0	18	IV quinine, 10 mg/kg every 8 h; IV clindamycin, 600 mg every 6 h
1	9	
2	2.4	IV quinine, 10 mg/kg every 8 h; IV clindamycin, 600 mg every 6 h; nasogastric atovaquone, 750 mg every 12 h
3	1.3	4 units red blood cells via manual exchange transfusion
4	0.9	
5	0.07	IV azithromycin, 250 mg every 24 h; IV clindamycin, 600 mg every 6 h; nasogastric atovaquone 750 mg every 12 h
6	0.1	
7	0.08	
8	0.05	IV doxycycline, 200 mg every 24 h
9	0.03	
10	0.03	
11	0.02	2 units of M- and S-antigen–negative red blood cells
12	0.01	
13	0.01	
14	0	
15	0	Antiparasitic agents discontinued

*IV, intravenous. †Percentage of parasitized erythrocytes detected by light microscopy of thin blood films.

The patient had no underlying immunosuppressive conditions and no history of splenectomy or reduced splenic function. She lived in a coastal town in Devon, UK; her only travel abroad in the preceding 12 months was a vacation in Tenerife, Spain, 5 months earlier. In the weeks before admission, she took walks along the coast, where cattle (the mammal host of *B. divergens*) grazed. Although ticks were in the area, she had not noticed any bites. She had no companion animals, but her daughter had a dog. She had never received blood products.

**Figure 2:**
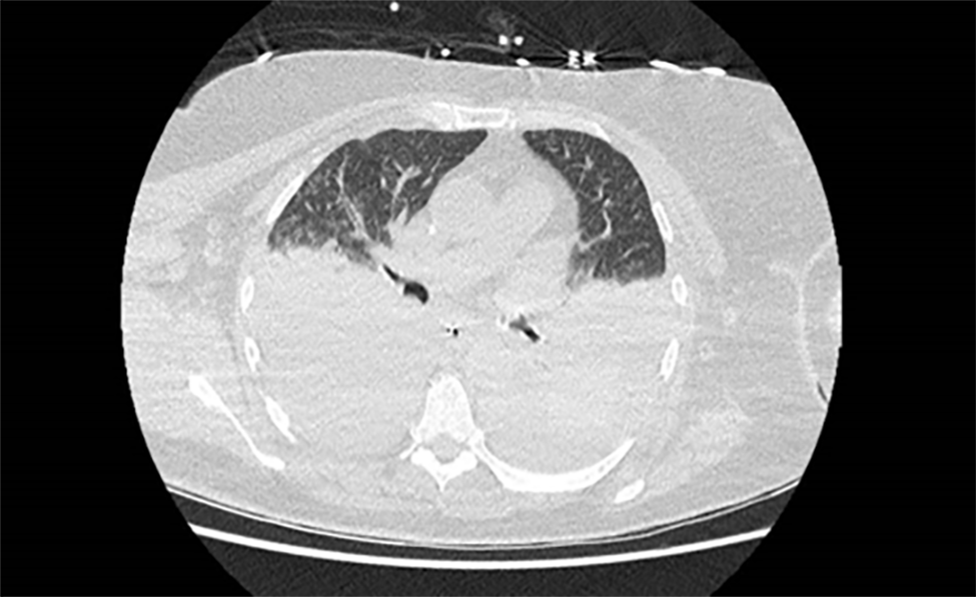
Axial computed tomography image from a case of autochthonous human *Babesia divergens* infection, England. Image shows bilateral pleural effusions.

**Figure 3:**
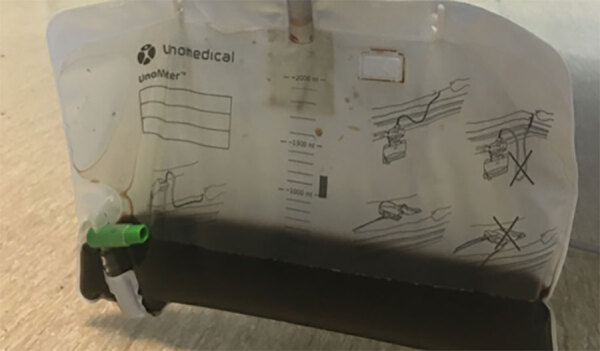
Urometer from a case of autochthonous human *Babesia divergens* infection, England. Black urine can be noted in the collection bag.

The patient’s illness was complicated by anuric acute kidney injury and fluid-refractory hypotension requiring renal dialysis and vasopressor support. Bilateral exudative pleural effusions developed (Figure 2), and she had hospital-acquired pneumonia. Severe intravascular hemolysis and black urine continued (Figure 3), which later showed evidence of hypersplenism, extravascular hemolysis, and thrombocytopenia. Her direct antiglobulin test remained negative throughout hospitalization. She underwent manual exchange transfusion with 4 units of O-negative blood and subsequent transfusion with 2 units of M- and S-antigen–negative erythrocytes on day 9 (Table). Because her recovery was protracted and thrombocytopenia was evolving, we added intravenous doxycycline (200 mg every 24 h) for possible *Anaplasma* co-infection.

Despite good parasitological response to treatment, the patient suffered a prolonged period of encephalopathy during convalescence. Magnetic resonance imaging of the brain showed an old cerebellar infarct, cerebrospinal fluid analysis was unremarkable, and an electroencephalogram showed nonspecific cerebral dysfunction.

Results of testing for immunoglobulins, complement blood tests, lymphocyte subsets, nuclear antibodies, tissue-transglutaminase antibodies, pneumococcal antibodies, and serum protein electrophoresis were all within reference ranges. Results of HIV serology and markers for hepatitis B and C viruses were negative; abdominal ultrasonography ruled out anatomic hyposplenia.

The patient’s encephalopathy gradually resolved, and she was extubated on day 13 of her ICU stay. Blood films on day 14 of treatment were negative, and antiparasitic agents were discontinued 24 hours later (Table). She subsequently recovered renal function and no longer required dialysis.

*B. divergens* infection was confirmed by 18S PCR and genomic sequencing from a blood sample drawn shortly after ICU admission (Appendix). Acute-phase serology was negative for tickborne encephalitis, *Rickettsia*, *Borrelia*, and *Anaplasma*. Convalescent serology for *Anaplasma* gave a weak, nonspecific reaction.

## Conclusions

*B. divergens*, transmitted by *Ixodes ricinus*, has been in England, including Devon, for >100 years (*9*). In mild bovine cases, babesiosis (also called Redwater fever) causes fever and anorexia; severe cases result in fatal hemolytic anemia with hemoglobinuria. This case documents emergence of autochthonous human babesiosis in England. Public health conducted a tick survey in the patient’s local area but found no ticks carrying *Babesia* spp. (*10*). Serologic surveys were not possible because *B. divergens* serology is unavailable in the United Kingdom; whether subclinical human *B. divergens* infections occurred in the locality at the time is unknown, but clinicians and veterinarians in England were notified of the case to raise awareness.

This case highlights the potential for severe *B. divergens* infection in the absence of depressed immunity. Severe *B. divergens* infection causes influenza-like illness and hemolysis, after which ≈40% of patients have multiorgan failure and die (*5*). In a case series in Europe, 84% of *B. divergens* infections were in patients with previous splenectomy (*3*). However, PCR-confirmed *B. divergens* infection was found in an immunocompetent adult in France, demonstrating the parasite’s ability to cause illness in previously healthy persons (*11*), as in our case. We considered whether *Anaplasma* co-infection increased illness severity in our patient, but that was not proven. For *B. microti*, patients with *B. burgdorferi* co-infection reportedly have more symptoms and longer illness than patients with either infection alone, although no uniform agreement exists between studies on co-infection, neither in humans nor animal models (*12*). Nevertheless, dual infections are increasingly seen, as have triple diagnoses with babesiosis, Lyme disease, and anaplasmosis (*13*). Thus, clinicians should suspect multi-infection in patients with an initial babesiosis diagnosis. 

Clinical laboratories diagnose *B. divergens* via blood film identification and PCR confirmation (*13*). Babesiosis treatment options include oral atovaquone and azithromycin in mild disease or intravenous clindamycin and quinine in severe cases (*13*). Extrapolating from *B. microti* treatment, oral atovaquone plus intravenous azithromycin is an option in *B. divergens* cases, but no randomized controlled trials or pharmacokinetic studies on *B. divergens* in humans are available. Exchange transfusion is recommended for parasitemia >10%, or moderate parasitemia with severe hemolytic anemia or organ dysfunction (*13*), and novel approaches to exchange transfusion have been suggested (*14*). No published trials are available, but to reduce parasitic invasion of additional erythrocytes in our patient, we administered 2 units of M- and S-antigen­–negative erythrocytes, which are resistant to *B. divergens* invasion, during the second transfusion (*14*).

In summary, the clinical and laboratory features of babesiosis and its rarity could lead clinicians to misdiagnose babesiosis as leptospirosis, urosepsis, or biliary sepsis and thus cause delays in appropriate therapy. *Babesia* also can be morphologically misidentified as *Plasmodium*. Furthermore, *Borrelia*, *Anaplasma*, or *Ehrlichia* co-infection can complicate the illness. Physicians should consider babesiosis as a differential diagnosis in patients with unexplained intravascular hemolysis, especially in rural areas.

Appendix Additional information on autochthonous human *Babesia divergens* infection, England.
