# Supplementary material for: Autochthonous Human Babesia divergens Infection, England
Source: Emerg Infect Dis. 2024 Oct;30(10):2165–8. doi: 10.3201/eid3010.240866 (PMC11431931; doi:10.3201/eid3010.240866)
Supplement: Appendix — Additional information on autochthonous human Babesia divergens infection, England. [file 24-0866-Techapp-s1.pdf]

Article DOI: https://doi.org/10.3201/eid3010.240866

EID cannot ensure accessibility for supplementary materials supplied by authors. Readers who have difficulty accessing supplementary content should contact the authors for assistance.

Autochthonous Human *Babesia divergens* Infection, England, England

Appendix

Additional Methods

EDTA treated patient blood (1 mL) (C2) and extracted DNA from patient blood (50 μL) (B2) were tested as follows: DNA was extracted from EDTA blood using the DNeasy Blood and Tissue Kit (Qiagen) following the manufacturer's instructions and DNA eluted in 200 mL buffer AE. Pan-piroplasm PCR was used following previously published conditions (*1*). Primers (Piro-A and Piro-B) were used at 10 pmol/mL and SYBR Green Jumpstart Ready Mix ready mix (Sigma**).** Amplification reactions were forty-five cycles of 94°C 15 seconds / 58°C 15 seconds / 72°C 30 seconds then a dissociation curve. Amplicons were checked on a 1.2% agarose gel impregnated with Sybr Safe and DNA visualized with UV illumination.

Amplicons were confirmed on an agarose gel with both samples from the patient producing a 400 bp (bp) amplicon of identical size to one produced from a DNA sample from a bovine case of *Babesia divergens* infection. The no template control produced no amplicon (Appendix Table).

Sequence Analysis

DNA from both patient samples and *B. divergens* control underwent sequencing with primers Piro A & Piro B. Both DNA samples produced a sequence of 364 bp and were 100% identical to APHA *B. divergens.* DNA sample B2 BLAST search produced results with 100% identity to *B. divergens* GenBank accession numbers MT550684, MG344781, MG344772, LC477143, LC477141, LC477139, MK510929, MG944238, KY296360, and KU748896. This species designation conforms with previous observations (*2*).

Sequence 2 (derived from DNA extracted from patients' blood sample) was submitted to NCBI GenBank with accession number PQ206416.

References

1. de Marco MDMF, Hernández-Triana LM, Phipps LP, Hansford K, Mitchell ES, Cull B, et al. Emergence of *Babesia canis* in southern England. Parasit Vectors. 2017;10:241. PubMed https://doi.org/10.1186/s13071-017-2178-5

2. Malandrin L, Jouglin M, Sun Y, Brisseau N, Chauvin A. Redescription of *Babesia capreoli* (Enigk and Friedhoff, 1962) from roe deer (*Capreolus capreolus*): isolation, cultivation, host specificity, molecular characterisation and differentiation from *Babesia divergens*. Int J Parasitol. 2010;40:277–84. PubMed https://doi.org/10.1016/j.ijpara.2009.08.008

Appendix Table. Real-time PCR results*

Well	Sample	Threshold	Ct (dR)	Dissociation Tm Product 1	Gel result
A2	No template control	495.004	No Ct	–	–
B2	Patient DNA	495.004	17.61	84.36	+
C2	Patient blood	495.004	17.23	83.90	+
D2	Horse blood control	495.004	No Ct	–	–
E2	*B. divergens*–positive (C2020)	495.004	21.30	84.4	+
F2	*B. motasi*–positive (Lamb #1)	495.004	42.90	84.88	ND
G2	*T. luwenshuni* positive (Lamb #2)	495.004	32.94	87.3	ND
H2	No template control	495.004	No Ct	–	ND

*Ct, cycle threshold; ND, not done; –, negative; +, positive.
